# Constitutively active c-Met kinase in PC-3 cells is autocrine-independent and can be blocked by the Met kinase inhibitor BMS-777607

**DOI:** 10.1186/1471-2407-12-198

**Published:** 2012-05-28

**Authors:** Yao Dai, Dietmar W Siemann

**Affiliations:** 1 Department of Radiation Oncology, University of Florida, 2033 Mowry Road, Cancer Genetic Research Complex, Room 485E, Gainesville, FL, 32610, USA

**Keywords:** BMS-777607, c-Met, HGF, Neutralizing antibody, Prostate cancer

## Abstract

**Background:**

The c-Met receptor tyrosine kinase is aberrantly activated in many solid tumors. In a prior study we showed that prostate cancer PC-3 cells exhibit constitutively activated c-Met without exogenous hepatocyte growth factor (HGF); however whether this characteristic is due to an endogenous HGF/c-Met autocrine loop remains controversial. In the current study we examined the response of PC-3 cells to an anti-HGF neutralizing antibody or a small molecule Met kinase inhibitor (BMS-777607).

**Methods:**

Cell scattering was tested by monitoring cell morphology after HGF stimulation. Cell migration was examined by both “wound-healing” and transwell assasy and invasion was detected by Matrigel-coated transwell assay. Proliferation, survival and anoikis were determined by MTT, colony formation and trypan blue exclusion assay, respectively. Gene and protein expression were assessed by real-time PCR and Western blot, respectively.

**Results:**

Although HGF mRNA could be detected in PC-3 cells, the molecular weight of secreted “HGF” protein was inconsistent with the functional recombinant HGF. Furthermore, conditioned medium from PC-3 cell cultures was ineffective at triggering either motogenic behavior or c-Met signaling in DU145, another prostate cancer cell line expressing c-Met but lacking basal c-Met activation. PC-3 cells also were not responsive to the anti-HGF neutralizing antibody in experiments assessing proliferation, migration, or c-Met signaling. BMS-777607 treatment with micromolar doses nonetheless led to significant inhibition of multiple PC-3 cell functions including proliferation, clonogenicity, migration and invasion. At the molecular level, BMS-777607 suppressed autophosphorylated c-Met and downstream c-Src and Akt pathways.

**Conclusions:**

These results suggest that the constitutive c-Met activation in PC-3 is independent of autocrine stimulation. Because PC-3 cells were responsive to BMS-777607 but not the anti-HGF antibody, the findings also indicate that under circumstances where c-Met is constitutively hyperactive in the absence of functional HGF, targeting the c-Met receptor remains a viable therapeutic option to impede cancer progression.

## Background

Oncogenic c-Met signaling is widely implicated in various human malignancies. Upon binding to its ligand, hepatocyte growth factor (HGF), the c-Met receptor initiates a signaling cascade leading to invasive growth and cancer cell dissemination [1,2]. In lung cancer, expression levels of both HGF and c-Met have been associated with advanced tumor stage and worse clinical outcome [3]. In prostate cancer, serum HGF has been identified as an independent prognostic factor for advanced disease [4,5] and c-Met expression in metastatic lesions frequently exceeds that of primary tumors, with positive expression reported in more than 90% of prostate cancer bone metastases [6,7].

The prevalence of the activation of the HGF/c-Met in human malignancies has driven rapid growth in drug development to target this signaling axis for cancer therapy. Strategies include antagonistic compounds, monoclonal antibodies, and small molecule kinase inhibitors [8]. Neutralizing antibodies targeting either HGF or c-Met have proven capable of impairing HGF-stimulated functions in either paracrine [9] or autocrine settings [10]. However, kinase inhibitors may have a broader range of application since Met kinase inhibitors may be efficacious in cancers driven by both HGF [11] and c-Met [12]. One leading candidate is ARQ197, a Met inhibitor that has shown activities in preclinical models and proves partial responses in patients with metastatic diseases [13]. BMS-777607 is another potent Met kinase inhibitor that entered clinical evaluation. Preclinical studies have shown that BMS-777607 delays the growth of human gastric cancer xenografts with MET gene amplification [14], inhibits HGF-induced metastasis-related functions in prostate cancer cells [15], and impairs pulmonary metastases in a rodent sarcoma model with hyperactivated c-Met [16]. These observations imply that BMS-777607 treatment may result in anti-proliferative and anti-metastatic effects in cancers with aberrant c-Met activity irrespective of the involvement of HGF.

Abnormal c-Met activation as a result of gene amplification, mutation, or transactivation can occur in certain cancer types [2]. However, c-Met overexpression due to upregulation at the transcriptional level remains the predominant event for the majority of human malignancies [17]. In this scenario, activation of the c-Met receptor still depends on the HGF ligand, however increased expression of c-Met on the cell surface could favor HGF-independent activation through spontaneous receptor dimerization [18]. In some cases, tumor cells express both HGF and c-Met, thus potentially establishing an autocrine loop in which the secreted HGF ligand by tumor cells binds to the c-Met receptor and causes its activation. Such HGF-dependent autocrine c-Met activation, considered a self-supportive mechanism for cell transformation, proliferation and survival, has been detected in various human primary and metastatic tumors, including breast cancer [19], glioma [20] and osteosarcoma [21].

Although prostate cancer PC-3 cells are responsive to exogenous HGF [15,22], our previous study showed that these cells exhibit a high basal level of autophosphorylated c-Met, suggesting that c-Met could be constitutively activated even in the absence of exogenous HGF [15]. However, whether such constitutive c-Met activation occurs in an autocrine manner is controversial. Some studies suggest the existence of an HGF/c-Met autocrine loop [18,23], whereas others indicate that PC-3 cells do not express HGF [24,25]. The current study examines the expression and function of HGF produced by PC-3 cells and the response of these cells to an anti-HGF neutralizing antibody or the small molecule Met kinase inhibitor, BMS-777607.

## Results

### HGF mRNA could be detected in PC-3 however secreted “HGF” is not consistent with the purified HGF protein

**Figure 1:**
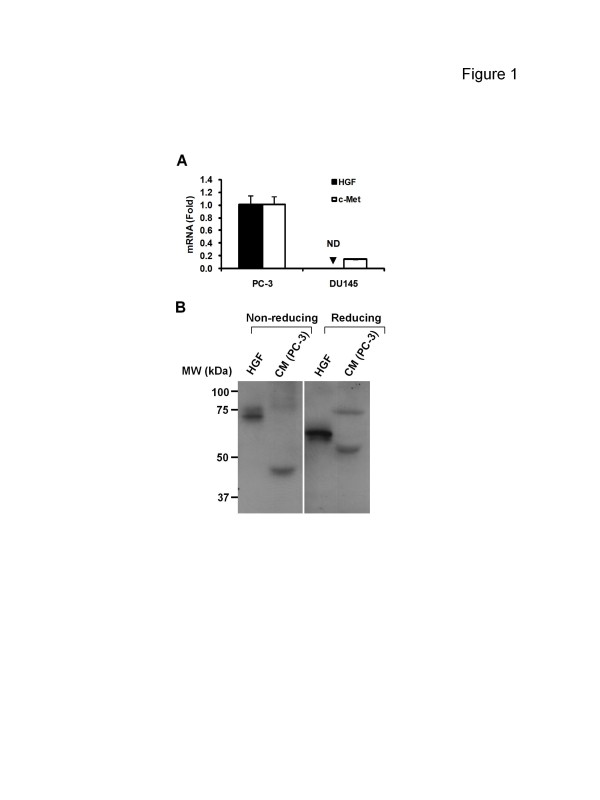
**Detection of HGF and c-Met in prostate cancer cellsA**, qPCR analysis of HGF and c-Met RNA levels in PC-3 and DU145 cells. Gene expression in PC-3 cells was defined as “1”. *Columns*, mean; *bars*, SD (*n* = 3). ND = not detectable. **B**, PC-3 cells were incubated in serum-free medium for 24 h. Condition medium (CM) was harvested and an aliquot amount (from 10^6^ cells) was analyzed by Western blot under both non-reducing and reducing conditions, with the recombinant human HGF (10 ng/lane) as a positive control. MW = molecular weight. kDa = kilo Dalton. Data are from 1 of 2 independent experiments.

We first tested the gene expression of both the HGF ligand and c-Met receptor in PC-3 and DU145 cells. HGF mRNA could be detected in PC-3 (C_T_ ~31.8) but not DU145 cells (C_T_ >37.0) (Figure 1A). In accordance with other studies [24], MET gene expression in PC-3 was higher than DU145 (Figure 1A, 7.1-fold). We next tested whether PC-3 cells secreted HGF protein by analyzing the CM. Mature HGF (~70-80 kDa) should contain a disulfide bond that can be cleaved in the presence of a reducing agent to generate an α (~ 60 kDa) and a β (~ 30 kDa) subunit [26]. As shown in Figure 1B, although the anti-HGF (α subunit) antibody could detect clear bands in the CM of PC-3 cells, the molecular weight of these bands did not match that of the α subunit of purified recombinant human HGF(~69 kDa and 60 kDa under non-reducing and reducing conditions, respectively).

### CM of PC-3 was not functional

**Figure 2:**
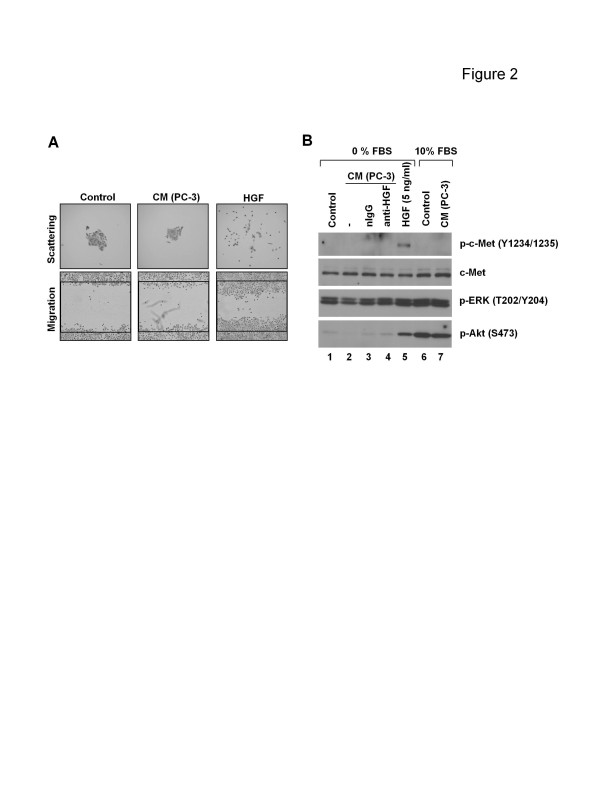
**Effect of anti-HGF neutralizing antibody on PC-3 cell functionsA**, Serum-starved DU145 cell colonies and scratched monolayers were incubated with CM (serum-free) from 10^6^ PC-3 cells or HGF (5 ng/ml) for 24 h, for the assessment of cell scattering and migration, respectively. Magnification, ×5. Data are from 1 of 3 independent experiments. **B**, serum-starved DU145 cells were incubated with CM (from 10^6^ PC-3 cells) in the absence (0%, lane 2) or presence of FBS (10%, lane 7), or pure exogenous HGF in a fresh FBS-free medium (5 ng/ml, lane 5) for 15 min, with untreated controls in a fresh medium with 0% (lane 1) or 10% FBS (lane 6) accordingly. In the CM with 0% FBS (lanes 2–4), DU145 cells were pre-treated with anti-HGF neutralizing antibody (anti-HGF, lane 4) or normal mouse IgG1 control (nIgG, lane 3) in a fresh serum-free medium at 37°C for 2 h, to confirm whether c-Met activation if could be triggered by the CM can be blocked by the neutralizing antibody. c-Met signaling molecules were analyzed by Western blot, with total c-Met as a loading control.

To determine whether the released “HGF” possessed biological function, serum-starved DU145 cells were incubated with CM from PC-3 cells. DU145 cells were used because these prostate cancer cells do not phosphorylate c-Met without exogenous HGF [27]. Unlike pure HGF, CM from PC-3 cells could not induce either scattering or migration in DU145 cells (Figure 2A). Furthermore, CM without serum (0% FBS) failed to induce phosphorylation of c-Met in the catalytic residues (Y1234/1235) and downstream molecules ERK (T202/Y204) and Akt (S473), which could be achieved by adding pure HGF (Figure 2B). To rule out the possibility that the secreted “HGF” may be inactivated in the absence of serum [28], CM with 10% FBS was tested. The results showed that c-Met was not phosphorylated by serum-containing CM (Figure 2B).

### PC-3 was not responsive to the anti-HGF neutralizing antibody

**Figure 3:**
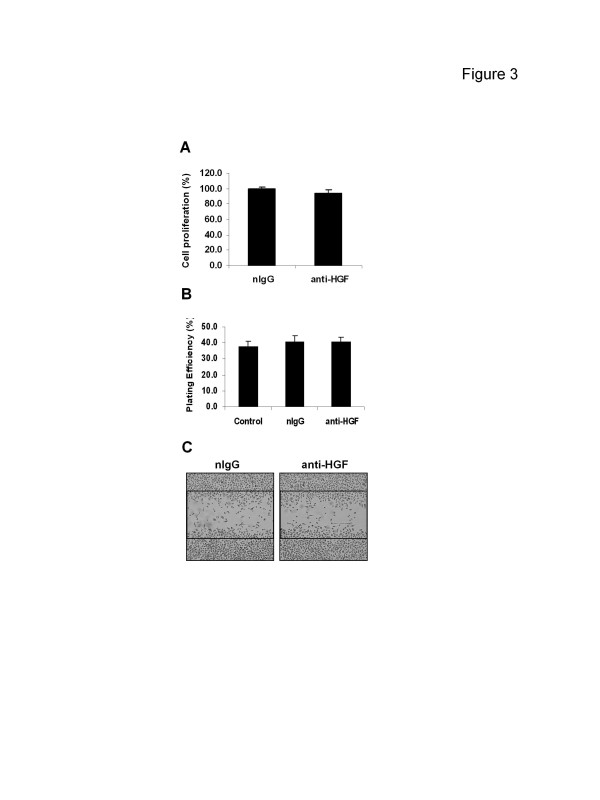
**Effect of anti-HGF neutralizing antibody on PC-3 cell functions** PC-3 cells were treated with 10 μg/ml of the neutralizing antibody (anti-HGF) or control antibody (nIgG) for 4 days (**A**), 14 days (**B**) or 1 day (**C**), for the detection of cell growth (MTT assay, **A**), clonogenicity (colony formation assay, **B**), or cell migration [“wound-healing” assay, **C** (magnification, ×5)]. *Columns*, mean; *bars*, SD (*n* = 6). Data represent ≥3 independent experiments.

The results of Figure 2 shown that CM from PC-3 cells cannot activate c-Met in DU145 cells; a cell line which does not express the HGF ligand but has the c-Met receptor (Figure 1A). To explore the functional effect of the secreted “HGF” on PC-3 cells themselves, cells were incubated with 10 μg/ml of an anti-HGF neutralizing antibody. This dose of the antibody, shown to be sufficient to neutralize HGF [9], did not reduce PC-3 cell proliferation (Figure 3A), colony formation (Figure 3B) or migration (Figure 3C), as compared to nIgG.

### Anti-HGF neutralizing antibody did not block constitutive c-Met signaling in PC-3

**Figure 4:**
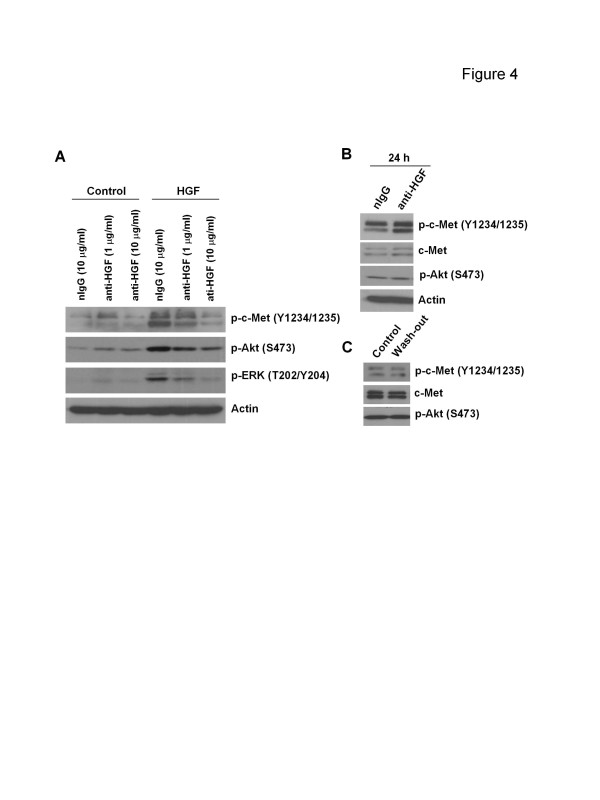
**Effect of anti-HGF neutralizing antibody on c-Met signaling in PC-3 cellsA**, PC-3 cells were serum-starved overnight and treated with the anti-HGF neutralizing antibody (anti-HGF) or control antibody (nIgG) for 2 h before stimulation with or without HGF (25 ng/ml) for 15 min. **B**, PC-3 cells were serum-starved over night and continuously treated with anti-HGF or nIgG for 24 h. **C**, PC-3 cells were serum-starved overnight and rinsed twice in a wash-out buffer (serum-free medium with 0.5 M NaCl, pH 3.7) for 30 s. Whole cell lysates were harvested immediately followed by Western blot analysis. Actin (**A**, **B**) or total c-Met (**C**) was used as a loading control.

To confirm that the anti-HGF antibody could block the c-Met pathway, PC-3 cells were incubated with the anti-HGF antibody under various conditions. Although phosphorylated c-Met and downstream targets such as Akt and ERK were suppressed by the anti-HGF antibody in a dose-dependent fashion in the presence of exogenous HGF, in the absence of HGF, these signaling molecules were not eliminated by the anti-HGF antibody as compared to nIgG (Figure 4A). Prolonged (24 h) treatment of the anti-HGF antibody also failed to decrease the basal level of p-c-Met and p-Akt in serum-deprived PC-3 cells (Figure 4B). To further exclude the possibility that the “HGF” that had been secreted before serum starvation could have bound the c-Met receptor and triggered constitutive c-Met phosphorylation, PC-3 cells were quickly rinsed with a wash buffer to strip any potential pre-existing “HGF” molecules on the cell surface [29]. The results showed that even after the rinse, the expression of p-c-Met and p-Akt still remained unchanged (Figure 4C).

### PC-3 was responsive to the small molecule Met kinase inhibitor BMS-777607

**Figure 5:**
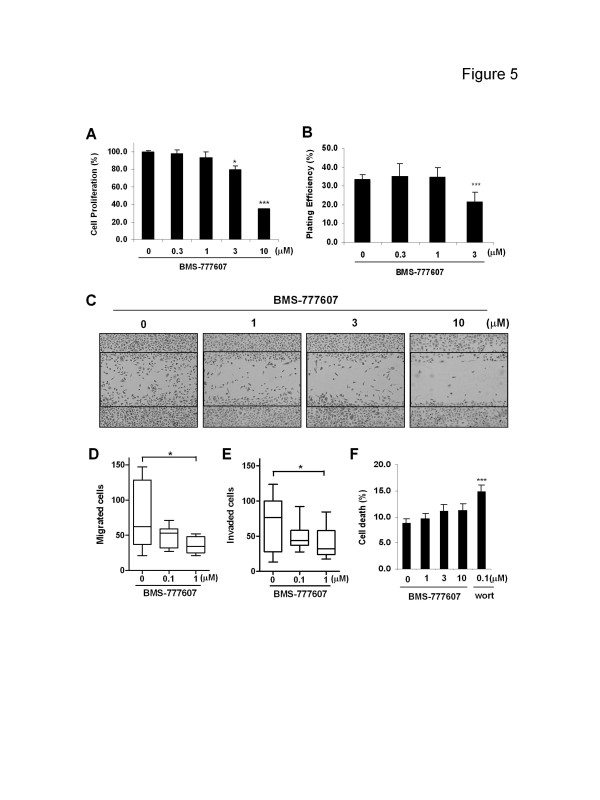
**Effect of Met inhibitor BMS-777607 on PC-3 cell functions** PC-3 cells were treated with indicated doses of BMS-777607 for 4 days (**A**), 14 days (**B**), 1 day (**C**), 2 days (**D**, **E**) and 3 days (**F**), for the detection of cell growth (MTT assay, **A**), clonogenicity (colony formation assay, **B**), cell motility [“wound-healing” assay, **C** (magnification, ×5)], migration (transwell assay, **D**), invasion (transwell assay, **E**) and anoikis (trypan blue exclusion assay, **F**), respectively. Data either represent 1 of 3 independent experiments (**B**, **C**) or are from > 3 independent experiments (**A**, **D**, **E**, **F**). **A**, **B**, **F**: *Columns*, mean; *bars*, SD (*n*≥9). **D**, **E**: *boxes*, median (middle line) with 75^th^ (upper boundary) and 25^th^ (lower boundary) percentiles; *whiskers*, 90^th^ (upper) and 10^th^ (lower) percentiles (*n* = 8). *, *P* < 0.05 (*t*-test in **A** and Wilcoxon test in **D**, **E**); ***, *P* < 0.001 (*t*-test in **A**, **B**, **F**). Wort = Wortmannin.

To test whether a small molecule Met kinase inhibitor could impair critical Met associated cell functions, PC-3 cells were exposed to BMS-777607. Both cell proliferation (Figure 5A, 20.5±3.9% inhibition at 3 μM; 64.8±0.1% inhibition at 10 μM) and clonogenicity (Figure 5B, 38.2±9.9% inhibition at 3 μM) were found to be impaired by BMS-777607 with doses greater than 1 μM. However, apoptosis was not observed even with the highest drug concentration (data not shown). Migration assessed using a “wound-healing” assay showed that this agent reduced the number of cells moving into the denuded area at concentrations ≥1 μM. Moreover, in the transwell-assays, both cell migration (Figure 5D) and invasion (Figure 5E) were found to be significantly inhibited by BMS-777607 at 1 μM (*P* < 0.05).

Anoikis is a mode of anchorage-independent cell death that negatively affects cancer cell dissemination and anoikis-resistance is considered as a critical player in prostate cancer metastasis [30-32]. To test whether Met inhibition will lead to anoikis, suspended PC-3 cells were incubated with BMS-777607 or wortmannin (an irreversible PI3K inhibitor) for 3 days. While wortmannin significantly increased anchorage-independent cell death (*P* < 0.001), BMS-777607 did not significantly affect anoikis even at the highest dose tested (10 μM, *P* = 0.13) (Figure 5F).

### BMS-777607 blocked constitutive c-Met signaling in PC-3 cells

**Figure 6:**
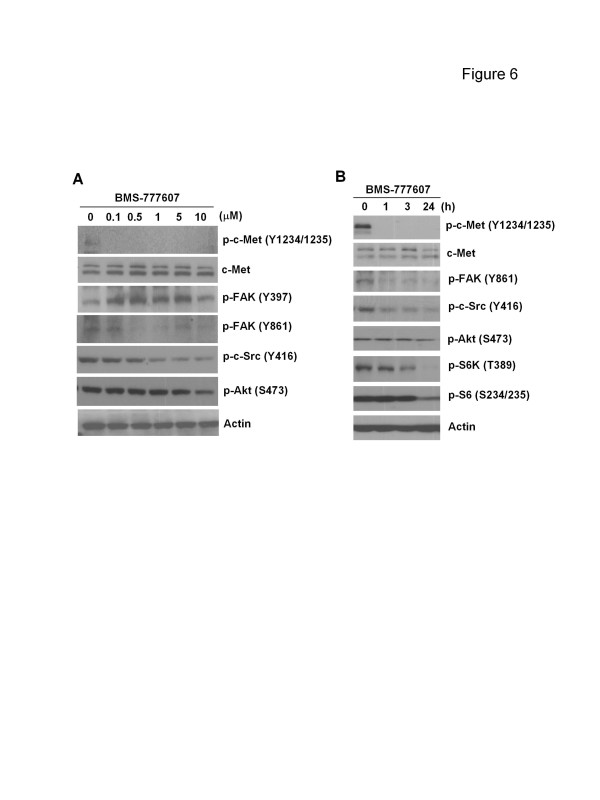
**Effect of BMS-777607 on c-Met signaling in PC-3 cells** PC-3 cells were treated with indicated doses of BMS-777607 for 1 h (**A**) or with BMS-777607 (1 μM) for indicated times (**B**). Whole cell lysates were harvested and analyzed by Western blot, with actin as a loading control. Data represent 1 of 2 independent experiments.

To investigate signaling alterations after c-Met kinase inhibition, cells were exposed to BMS-777607 for various doses and times. BMS-777607 completely eliminated c-Met autophosphorylation at doses as low as 0.1 μM (Figure 6A). While p-Akt was modestly inhibited by BMS-777607 at the highest dose (10 μM), expression levels of autophosphorylated Src (Y416) and Src-dependent phosphorylated FAK (Y861) were decreased with doses greater than 0.5 μM (Figure 6A). In contrast, autophosphorylated FAK (Y397) was not affected by BMS-777607 (Figure 6A). When cells were treated with BMS-777607 for prolonged periods (24 h), phosphorylation of c-Met, c-Src and FAK (Y861) remained inhibited. Furthermore, phosphorylation of Akt and mammalian target of rapamycin (mTOR) as well as downstream molecules S6K (T389) and S6 (S234/235) started to be ablated at 3 ~ 24 h after drug treatment (Figure 6B). ERK phosphorylation however, showed little change by either high dose or long term treatment (data not shown).

## Discussion

MET oncogene overexpression has been described in a variety of human cancers including prostate [7]. Aberrant c-Met activation has been shown to be strongly involved in prostate cancer aggressiveness and poorly clinical outcome [2,6,7]. In the current study human metastatic prostate cancer PC-3 cells were found to overexpress not only c-Met but also HGF at the transcriptional level (Figure 1A). Since a high basal level of phosphorylated c-Met is also observed in PC-3 cells, it was anticipated that an HGF/c-Met autocrine loop that induces constitutive c-Met activation exist in this cell line. However, the molecular weight of the secreted “HGF” by PC-3 cells was inconsistent with the recombinant HGF protein (Figure 1B). Furthermore, c-Met-associated functions were not activated by CM from PC-3 cells (Figure 2), suggesting that what was secreted by these cells was not functional HGF. This conclusion was subsequently supported by evidence indicating that PC-3 cells did not respond to the anti-HGF neutralizing antibody (Figures 3 and 4); a finding that supports the conclusion that the constitutive c-Met activity in PC-3 cells is autocrine-independent.

Two questions arise from the results of the current study. Firstly, what is the “HGF” produced by PC-3 cells and what is its function? Mature HGF/SF is composed of an α-chain (69 kDa) and a β-chain (34 kDa) that are linked to form a heterodimer [33]. Since the primers are designed to probe the α subunit of HGF mRNA (Figure 1A) and a single band (~ 45 kDa) can be detected under non-reducing conditions (Figure 1B, left), the secreted protein might be an isoform of HGF [34]. Secondly, if an autocrine loop is not involved, then what accounts for the constitutive c-Met activation? To date MET gene abnormalities such as activating mutations or amplifications have not been reported in PC-3 cells nor prostate cancer in general [2], suggesting alterations at the genetic level may not be involved. Since c-Met protein overexpression due to mRNA upregulation occurs predominantly in human cancers [17], the basal level of phosphorylated c-Met in PC-3 cells may simply be a result of increased MET transcripts via unknown mechanisms. In addition, the cross-talk between c-Met and other signaling molecules post-transcriptionally could be a possibility given that c-Met is able to be transactivated by several other transmembrane proteins [35]. In the PC-3 cell line, basal c-Met phosphorylation remained unaffected by exposure to either gefitinib or dasatinib (data not shown), suggesting that c-Met is not activated by epidermal growth factor receptor (EGFR) or c-Src, two kinases shown to be involved in c-Met transactivation in some studies [36,37]. However other signaling molecules such as Ron, another Met receptor family member which is also overexpressed in PC-3 cells [38], might transactivate c-Met. Finally, an HGF-mediated “intracellular” autocrine mechanism [25], although rare, could be another possibility.

Despite the unresponsiveness of PC-3 cells to anti-HGF antibody, the Met kinase inhibitor BMS-777607 did significantly inhibit PC-3 cell proliferation, clonogenicity, migration and invasion (Figure 5) as well as c-Met signaling pathways (Figure 6). Coupled with our previous findings [15], these results suggest that in the PC-3 tumor model, c-Met signaling plays a major role in the metastasis-related behavior irrespective of the HGF status. Consistent with the impact on cellular functions, BMS-777607 also significantly ablated molecular c-Met activity and downstream pathways including c-Src/FAK and Akt-mTOR, indicating that c-Src and Akt are two mediators of constitutive c-Met signaling. Interestingly, exogenous HGF cannot phosphorylate c-Src in PC-3 cells, suggesting that c-Src does not mediate HGF-induced c-Met activation [15]. The discrepant role of c-Src in c-Met-mediated molecular events reveals the complex interplay between these signaling components.

PC-3 cells were originally isolated from a prostate cancer bone metastasis [39]. Since HGF is enriched in the stroma of both the prostatic gland [40] and bone marrow [41] and is considered to be sufficient to trigger c-Met activation, acquisition of the c-Met activity in the absence of environmental HGF may facilitate tumor cells to survive and metastasize in a scenario where exogenous HGF is lacking. Anchorage independence is suggested as a factor in the survival of circulating tumor cells [42], but our data indicate that c-Met is not essential for maintaining anchorage-independent cell survival (Figure 5F). Thus while targeting c-Met kinase is unlikely to reduce viability of non-adherent tumor cells, small molecule Met kinase inhibitors may have significant therapeutic potential as agents that interfere with the metastatic phenotypes associated with c-Met.

## Conclusions

In summary, the current study showed that the Met kinase inhibitor BMS-777607, but not the anti-HGF neutralizing antibody, exerted suppressing effects on c-Met-associated cellular functions in PC-3 cells that express constitutively activated c-Met. These findings suggest the possibility that in cancers where hyperactive c-Met is independent of HGF-mediated autocrine stimulation, targeting the Met receptor may be more effective than targeting HGF ligand to impede cancer progression and metastasis.

## Methods

### Reagents and antibodies

BMS-777607 was kindly provided by Dr. Joseph Fargnoli (Bristol-Myers Squibb, Piscataway, NJ). The powder was dissolved in dimethyl sulfoxide (DMSO) and stored at −20°C. Recombinant human HGF, anti-HGF neutralizing antibody and normal mouse IgG1 isotype control were purchased from R&D Systems (Minneapolis, MN). Wortmannin was obtained from Calbiochem (Gibbstown, NJ). Additional chemicals were purchased from Sigma (St. Louis, MO) unless otherwise indicated. The following primary antibodies were used: phospho-c-Met (Y1234/1235), total c-Met, phospho-Akt (S473), phospho- extracellular signal-regulated kinases (ERK, T202/Y204), phospho-c-Src (Y416), phospho-focal adhesion kinase (FAK, Y861), phospho-S6 kinase (S6K, T389) and phospho-S6 (S234/235) (Cell Signaling, Danvers, MA); phospho-FAK (Y397) (Chemicon, Billerica, MA); β-actin (Sigma); HGF (only recognizes α subunit of the HGF protein, Santa Cruz, Santa Cruz, CA).

### Cell culture

Human prostate cancer cell lines PC-3 and DU145 were obtained from the American Type Culture Collection. PC-3 and DU145 cells were maintained in Ham’s F-12 K and DMEM respectively, supplemented with 10% fetal bovine serum (FBS), 100 U/ml penicillin, and 100 μg/ml streptomycin. Cells were cultured in a 5% CO_2_ humidified incubator at 37°C. All experiments were performed using cells in 10 passages.

### Conditioned medium (CM)

Subconfluent PC-3 cells were incubated with complete or serum-free medium for 24 h. The supernatant was collected and spun down at 2,500 rpm for 10 min to remove any intact cells or cell debris. CM was further concentrated by centrifuging at 2,000 × g for 20 min using an Amicon Ultra Centrifugal filter device (cut-off molecular weight 10 kDa, Millipore). CM from 10^6^ cells was analyzed by Western blot on a 10% SDS-polyacrylamide gel under both reducing (with β-mercaptoethanol) and non-reducing (without β-mercaptoethanol) conditions to detect secreted HGF. In some circumstances, CM was directly used for the experiments without concentration.

### Cell scattering

Cells were seeded in a 6-well plate (10^3^ cells/well) and cultured for 7 days until colonies formed. Cell colonies were incubated with serum-free medium overnight and challenged with either CM or pure HGF (5 ng/ml). Cells were stained with crystal violet (0.1%) 24 h after treatment. Scattered colonies were photographed.

### Cell proliferation

Cells were seeded in a 96-well plate at a density of 5 × 10^3^ cells/well and exposed to desired agents for a period of 96 h. At the end of the treatment period cells were incubated with WST-8 (MTT assay reagent) in a Cell Counting Kit (Dojindo Molecular Technologies, Rockville, MD) according to the manufacturer’s instruction. Absorbance was determined at 450 nm colorimetrically. Cell proliferation (%) was calculated as the ratio of the absorbance from treated samples compared to that of the untreated control sample.

### Colony formation

Cells were seeded into a 6-well plate and continuously exposed to desired agents for 14 days. Plates were stained with crystal violet and cell colonies (> 50 cells) were counted. Plating efficiency was calculated as the percentage of seeded tumor cells forming macroscopic colonies.

### Cell migration

Cell migration was determined using both “wound-healing” and transwell assays. For the “wound-healing” assay, cells were seeded in a 6-well plate and grown for 48 h to allow them to reach confluency. Prior to the treatment, a 2 mm wide scratch was made in the monolayer using a sterilized 1 ml pipette tip. Cell migration was assessed 24 h after treatment. For the transwell assay, cells (10^4^/insert) were seeded into a commercial transwell insert (8 μm pore membrane) and incubated with desired agents. Migrated cells on the bottom of the filter were stained and counted under a light microscope 24 h after treatment.

### Cell invasion

Invasive ability of cells was tested using a transwell insert pre-loaded with Matrigel (BD Biosciences, San Diego, CA). Inserts were incubated with serum-free medium at 37°C for 2 h to allow rehydration of Matrigel. Agents to be tested were added into both upper and lower chambers at equal concentrations. Cells suspended in serum-free medium were then loaded onto the top chamber (10^4^/insert). Complete medium was used in the lower chamber as a chemo-attractant. After 24 h of incubation, the Matrigel was removed and the inserts were stained with crystal violet. Invaded cells on the underside of the filter were counted.

### Anoikis

Cells were seeded into a 6-well plate coated with poly-HEMA (2-hydroxyethyl methacrylate, 2 mg/100 μl/cm^2^) at a density of 10^5^/well and continuously incubated with the compounds for 72 h. The suspended cells were harvested and incubated with trypsin-EDTA (0.05%) at 37°C for 20 min to dissociate cell clumps. Single cell suspensions were stained with the trypan blue and cells were counted using a hemocytometer. Cell death (%) was calculated from the ratio of positive-stained to total cells.

### Western blot

Cells were harvested and disrupted in a radioimmunoprecipitation assay (RIPA) lysis buffer buffer (50 mM Tris–HCl, pH 8.0, 150 mM NaCl, 0.1% SDS, 1% NP-40, 0.25% sodium deoxycholate and 1 mM EDTA with protease inhibitor cocktail, 1 mM NaF and 1 mM Na_3_VO_4_). Equal amounts of whole cell lysates were resolved by SDS-PAGE (Bio-Rad), electrotransferred to a nitrocellulose membrane (Bio-Rad), probed with relevant primary antibodies at 4°C overnight, incubated with horseradish peroxidase (HRP) conjugated secondary antibodies (Jackson ImmunoResearch, West Grove, PA) and detected with an enhanced chemiluminescence substrate (Amersham, Piscataway, NJ).

### Quantitative real time PCR (qPCR)

qPCR was performed as described previously [43]. Briefly, total RNA was extracted using TRIzol (Invitrogen) and reverse transcription was conducted following the instructions of the TaqMan Reverse Transcription Kit (Applied Biosystems, Foster City, CA). For qPCR, 1 μl gene primers with SYBR Green PCR Master Mix (Applied Biosystems) in 20 μl reaction volume was performed. Primers were designed as: HGF, forward, 5′-CTCACACCCGCTGGGAGTAC-3′, reverse, 5′-TCCTTGACCTTGGATGCATTC-3′; c-Met, forward, 5′-CTGCCTGCAATCTACAAGGT-3′, reverse, 5′-ATGGTCAGCCTTGTCCCTC-3′; actin (an internal control): forward, 5′-CTCCTCCTGAGCGCAAGTACTC-3′, reverse, 5′-TCCTGCTTGCTGATCCACATC-3′. All reactions were performed on the ABI7500 Fast Real-Time PCR System (Applied Biosystems). mRNA levels of tested genes were normalized to Actin according to the following formula: 2^ –(C_T_ test – C_T_ Actin), where C_T_ is the threshold cycle. Fold of gene expression of PC-3 cells was defined as “1”.

### Statistical analysis

Two-tailed Student’s *t*-test (parametric) or Wilcoxon rank sum test (non-parametric) were employed for data analysis by GraphPad Prism 5.0 (San Diego, CA). A threshold of *P* < 0.05 was defined as statistically significant.

## Abbreviations

HGF, Hepatocyte growth factor; DMSO, Dimethyl sulfoxide; ERK, Extracellular signal-regulated kinases; FAK, Focal adhesion kinase; S6K, S6 kinase; FBS, Fetal bovine serum; CM, Conditioned medium; poly-HEMA, 2-hydroxyethyl methacrylate; RIPA, Radioimmunoprecipitation assay; HRP, Horseradish peroxidase; mTOR, Mammalian target of rapamycin.

## Competing interests

The authors declare that they have no competing interests.

## Authors’ contributions

YD conceived of the study, carried out all the experiments and drafted the manuscript. DWS participated in the design of the study and revised the manuscript. Both authors read and approved the final manuscript.

## Pre-publication history

The pre-publication history for this paper can be accessed here:

http://www.biomedcentral.com/1471-2407/12/198/prepub
